# DNA methylation and the epigenetic clock in relation to physical frailty in older people: the Lothian Birth Cohort 1936

**DOI:** 10.1186/s13148-018-0538-4

**Published:** 2018-08-03

**Authors:** Catharine R. Gale, Riccardo E. Marioni, Sarah E. Harris, John M. Starr, Ian J. Deary

**Affiliations:** 1 MRC Lifecourse Epidemiology Unit, Southampton General Hospital, University of Southampton, Southampton, SO16 6YD UK; 2 Centre for Cognitive Ageing and Cognitive Epidemiology, University of Edinburgh, 7 George Square, Edinburgh, EH8 9JZ UK; 3 Department of Psychology, University of Edinburgh, Edinburgh, EH8 9JZ UK; 4 Medical Genetics Section, Centre for Genomics and Experimental Medicine, Institute of Genetics and Molecular Medicine, University of Edinburgh, Edinburgh, EH4 2XU UK; 5 Alzheimer Scotland Dementia Research Centre, University of Edinburgh, Edinburgh, EH8 9JZ UK

**Keywords:** Frailty, Aging, Epigenome-wide association study, Epigenetic age acceleration

## Abstract

**Background:**

The biological mechanisms underlying frailty in older people are poorly understood. There is some evidence to suggest that DNA methylation patterns may be altered in frail individuals.

**Methods:**

Participants were 791 people aged 70 years from the Lothian Birth Cohort 1936. DNA methylation was measured in whole blood. Biological age was estimated using two measures of DNA methylation-based age acceleration-extrinsic and intrinsic epigenetic age acceleration. We carried out an epigenome-wide association study of physical frailty, as defined by the Fried phenotype. Multinomial logistic regression was used to calculate relative risk ratios for being physically frail or pre-frail according to epigenetic age acceleration.

**Results:**

There was a single significant (*P* = 1.16 × 10–7) association in the epigenome-wide association study comparing frail versus not frail. The same CpG was not significant when comparing pre-frail versus not frail. Greater extrinsic epigenetic age acceleration was associated with an increased risk of being physically frail, but not of being pre-frail. For a year increase in extrinsic epigenetic age acceleration, age- and sex-adjusted relative risk ratios (95% CI) for being physically frail or pre-frail were 1.06 (1.02, 1.10) and 1.02 (1.00, 1.04), respectively. After further adjustment for smoking and chronic disease, the association with physical frailty remained significant. Intrinsic epigenetic age acceleration was not associated with physical frailty status.

**Conclusions:**

People who are biologically older, as indexed by greater extrinsic epigenetic age acceleration, are more likely to be physically frail. Future research will need to investigate whether epigenetic age acceleration plays a causal role in the onset of physical frailty.

**Electronic supplementary material:**

The online version of this article (10.1186/s13148-018-0538-4) contains supplementary material, which is available to authorized users.

## Introduction

Frailty is a clinical syndrome that becomes increasingly common at older ages [1]. Its core features are increased vulnerability to stressors due to impairments in multiple systems, decreased physiological reserves, and a decline in the ability to maintain homeostasis [2]. It increases the risk of adverse outcomes, including falls, disability, hospitalization, institutionalization, and death [2–4]. There are two principal models of frailty [2]. Fried’s phenotype model defines frailty on purely physical terms, based on three or more components (poor grip strength, slow walking speed, low physical activity, exhaustion, and unintentional weight loss) [3]. The frailty index, or cumulative deficit model, defines frailty much more broadly in terms of the accumulation of ‘deficits’ (symptoms, signs, diseases, and disabilities) [5]. The biological drivers of the multisystem dysregulation that underlies frailty remain unclear, particularly at the cellular and molecular levels.

Epigenetic changes affect all cells and tissues over the lifespan [6]. One such change involves alterations to deoxyribonucleic acid (DNA) methylation patterns. DNA methylation is involved in the regulation of gene expression and occurs at cytosine-phosphate-guanine (CpG) sites across the genome [7, 8]. The proportion of methylation at a particular CpG site is referred to as a beta value, which can change over the life-course [7, 8]. Methylation levels are affected by both genetic and environmental exposures [9]. The relationship between DNA methylation levels and aging is complex [10]. Early evidence showed that global DNA methylation level decreases with age, but subsequent studies revealed that aging is associated with differential methylation (mainly hypermethylation) of some genomic loci [11]. Multiple CpG sites have been identified where methylation is associated with age [12]. Several DNA methylation-based biomarkers are now used to estimate ‘epigenetic age’ [13, 14] or ‘epigenetic age acceleration,’ a measure of the difference between predicted epigenetic age and chronological age’ [15]. These indices—often referred to as the ‘epigenetic clock’—are associated with mortality independently of chronological age and other risk factors, supporting the notion that they capture some aspect of biological aging [15, 16]. Epigenetic alterations are thought to be one of the ‘hallmarks’ of aging [10] and hence may contribute to age-related pathologies such as frailty. That is, chronological age acts as a proxy for several biological changes associated with aging, of which DNA methylation is one.

Few studies have investigated the relationship between DNA methylation patterns and frailty in older people. One study, where frailty status was defined using cluster analysis, reported that global DNA methylation was lower in people who were frail compared to the non-frail, [17], but another study using the Fried phenotype of physical frailty found no such association [18]. This latter study also examined associations between promotor-specific CpG island methylation and frailty status, and found that lower levels of CpG island methylation were associated with a reduced likelihood of being frail [18]. Further indications that DNA methylation patterns might differ in people who are frail came in a recent study of 1820 older people which found that greater epigenetic age acceleration—the difference between predicted epigenetic age and chronological age—was associated with greater frailty as measured by a broadly defined frailty index, such that the frailty index increased by about 0.25% points per year of epigenetic age acceleration [19].

Here, we aimed to add to understanding of the relationship between epigenetic status and physical frailty in a large, narrow-age sample of 70-year olds. The limited chronological age range mitigates against cohort exposure effects on DNA methylation thus providing a more robust context to draw inferences about biological aging indices. First, we conducted an epigenome-wide association study (EWAS) to try to identify whether differential methylation at specific CpG sites was associated with current physical frailty status, as defined by the Fried phenotype. Secondly, we investigated whether people who were biologically older as indexed by epigenetic age acceleration measures were at increased risk of being physically frail.

## Methods

### Participants

The Lothian Birth Cohort 1936 (LBC1936) was established to study cognitive aging in surviving members of the 1947 Scottish Mental Survey [20, 21]. 1091 community-dwelling people were recruited aged around 70 years, mostly from the Edinburgh area of Scotland. This was wave 1 of the LBC1936, data from which are used in the present study. Ethical approval was obtained from the Multi-Centre Ethics Committee for Scotland and Lothian Research Ethics Committee. All subjects provided written informed consent.

### DNA methylation and epigenetic age acceleration measures

Whole blood DNA methylation was measured using the Illumina HumanMethylation450BeadChips [8] in 1004 participants from samples collected at mean age 70 years. Methodological details about collection of the methylation data and quality control processes have been reported previously [9, 22]. Briefly, data were available on 485,512 CpGs in 920 participants after quality control. This included background correction, the removal of probes with a low detection rate, low-quality samples and samples with a low call rate, and samples where there was a sex or genotype mismatch. These probes were then used to calculate two measures of epigenetic age. Calculation of these measures was done online at https://labs.genetics.ucla.edu/horvath/dnamage/. First, the epigenetic age of each participant was estimated from their blood sample in two ways, using the approaches of Horvarth [13] and Hannum [14]. Intrinsic epigenetic age acceleration (IEAA) was then defined as the residuals from a linear regression analysis of Horvarth’s estimate of epigenetic age on chronological age and blood immune cell counts (plasmablasts, naive, and exhausted CD8+ T cells, CD4+ T cells, natural killer cells, monocytes, and granulocytes) imputed from methylation data. IEAA is therefore independent of chronological age and much of the variation in blood cell composition. IEAA is intended to capture cell-intrinsic properties of the aging process. Extrinsic epigenetic age acceleration (EEAA) was calculated by calculating a weighted average of Hannum’s estimate of epigenetic age and three immune blood cell types known to change with age, as described in Chen et al. [15], and then saving the residuals from a linear regression analysis of the resulting epigenetic age estimate on chronological age. EEAA tracks both age-related changes in blood cell composition and intrinsic epigenetic changes [15]. Like IEAA, EEAA is independent of chronological age.

### Physical frailty

Physical frailty status was assessed during the LBC1936 wave 1 (mean age 70 years) survey using the Fried frailty phenotype [3]. Frailty is defined as the presence of three or more of the following components: weakness, self-reported exhaustion, slow gait speed, unintentional weight loss, and low physical activity. Pre-frailty is defined as the presence of one or two of these components.

Maximum handgrip strength was measured three times on each side using a dynamometer; the best of these measurements was used for analysis. Body mass index (BMI) was calculated as weight (in kilograms)/height (in meters)^2^. Gait speed was assessed by measuring time taken to walk 6 m at maximum speed. Participants were asked to indicate their usual level of physical activity on a 6-point scale, ranging from ‘moving only in connection with necessary (household) chores’ to ‘keep-fit/heavy exercise or competitive sport several times a week.’ Symptoms of depression were assessed using the depression subscale of the Hospital Anxiety and Scale (HADS-D) [23]. We operationalized the frailty components using definitions similar to those used in Fried’s original studies [3, 24]: weakness was defined as maximum grip strength in the lowest 20% of the distribution, taking account of sex and BMI; exhaustion was considered present if the participant responded positively to the HADS-D question ‘I feel as if I’m slowed down’; slow gait speed was defined as a walking speed in the lowest 20% of the distribution, taking account of sex and height; as no information was available on loss of weight prior to recruitment, we considered participants to have unintentional weight loss if they had a current BMI < 18.5 kg/m^2^, as has been done previously; [24] low physical activity was defined as activity in the lowest sex-specific 20% of the distribution.

### Covariates

In addition to age and sex, we used white blood cell counts as covariates. These are associated with DNA methylation levels, [25, 26] and were measured in the same blood sample. Five cell types were assessed: basophils, monocytes, lymphocytes, eosinophils, and neutrophils. For measurement details see McIllhagger et al. [27]. When examining the relationship between epigenetic age acceleration measures and physical frailty status, we also adjusted for smoking status (categorized as never smoked, ex-smoker, current smoker), units of alcohol consumed per week, and number of chronic physical diseases present. Participants provided information during interview on whether they had been diagnosed with diabetes, stroke, cardiovascular disease, high blood pressure, arthritis, or cancer. We summed the number of chronic physical conditions present as an indicator of morbidity burden. This simple measure is a common way of ascertaining morbidity burden, [28] and has been shown to be almost as effective at predicting mortality and health care costs as more complex methods [29].

### Statistical analyses

Epigenome-wide association study analyses were conducted whereby each methylation CpG was regressed on the Fried frailty phenotype (treated as a factor with ‘not frail’ as the reference category) using linear regression, adjusting for age, sex, and white blood cell counts. A Bonferroni *p* value threshold (0.05/485, 512) was set.

We used multinomial logistic regression to derive relative risk ratios for being physically frail or pre-frail per year increase in extrinsic and intrinsic epigenetic age acceleration. Estimates are shown adjusted for age and sex, then further adjusted for smoking status, alcohol intake, and number of chronic physical diseases. Sensitivity analyses were carried out with adjustments for white cell counts and technical measures related to DNA methylation typing, namely sample plate, BeadChip, position on BeadChip, and date.

### Analytical sample

Of the 1091 participants who took part in the wave 1 survey, 953 (87.3%) had data on the five components that are used to derive the Fried phenotype of physical frailty, and 791 of these (83%) had methylation data after quality control. The analyses below are based on these 791 participants.

## Results

Characteristics of the 791 participants in the study sample are presented in Table 1 according to frailty status. Their mean age was 70.0 years (SD 0.84). In total, 7.8% of the participants were physically frail and 46.0% were pre-frail as defined by the Fried phenotype. Compared to those who were not frail, participants who were frail had a higher mean extrinsic epigenetic age acceleration, higher mean levels of two types of white cells, monocytes and neutrophils, more chronic physical illnesses, and a higher proportion of them were current smokers. Median weekly alcohol consumption was significantly lower in those who were frail than in those who were not frail.

**Table 1:** Characteristics of the study participants according to physical frailty status

Characteristics	Total	Not frail (*n* = 365)	Pre-frail (*n* = 364)	Frail (*n* = 62)	*p* value for difference between not frail and frail
Age (years), mean (SD)	69.5 (0.84)	69.4 (0.88)	69.6 (0.81)	69.5 (0.70)	0.61
Female, number (%)	398 (50.3)	184 (50.4)	181 (49.7)	33 (53.2)	0.17
Epigenetic clock measures (years), mean (SD)					
Extrinsic epigenetic age acceleration	− 0.39 (7.11)	− 1.03 (7.55)	− 0.08 (6.73)	1.50 (6.12)	0.013
Intrinsic epigenetic age acceleration	− 0.45 (5.99)	− 0.78 (6.34)	− 0.23 (5.68)	0.20 (5.65)	0.254
Number of chronic physical illnesses, median (IQR)	1 (0–2)	1 (0–1)	1 (0–2)	2 (1–2)	0.0001
Smoking status, number (%)					
Never	374 (47.3)	182 (49.9)	168 (46.2)	24 (38.7)	0.005
Ex-smoker	334 (42.2)	154 (42.2)	155 (42.6)	25 (40.3)	
Current smoker	83 (10.5)	29 (7.95)	41 (11.3)	13 (21.0)	
Units of alcohol per week, median (IQR)	6 (0.5–14)	1 (0–7)	4.25 (0.5–14)	0 (0.25–10)	0.009
White blood cell counts (10^9^/L), median (IQR)					
Basophils	0.04 (0.03–0.05)	0.04 (0.03–0.05)	0.04 (0.03–0.05)	0.04 (0.03–0.06)	0.096
Eosinophils	0.13 (0.08–0.21)	0.12 (0.07–0.20	0.12 (0.08–0.22)	0.14 (0.08–0.24)	0.204
Monocytes	0.49 (0.40–0.61)	0.48 (0.38–0.58)	0.52 (0.42–0.63)	0.51 (0.45–0.61)	0.034
Lymphocytes	1.73 (1.40–2.15)	1.68 (1.37–2.05)	1.77 (1.41–2.27)	1.75 (1.48–2.23)	0.105
Neutrophils	4.42 (3.29–5.27)	4.09 (3.23–5.03)	4.29 (3.31–5.36)	4.60 (3.63–5.89)	0.007

The correlation between the two measures of epigenetic age acceleration was moderate (*r* = 0.38, *p* < 0.0001).

**Fig. 1:**
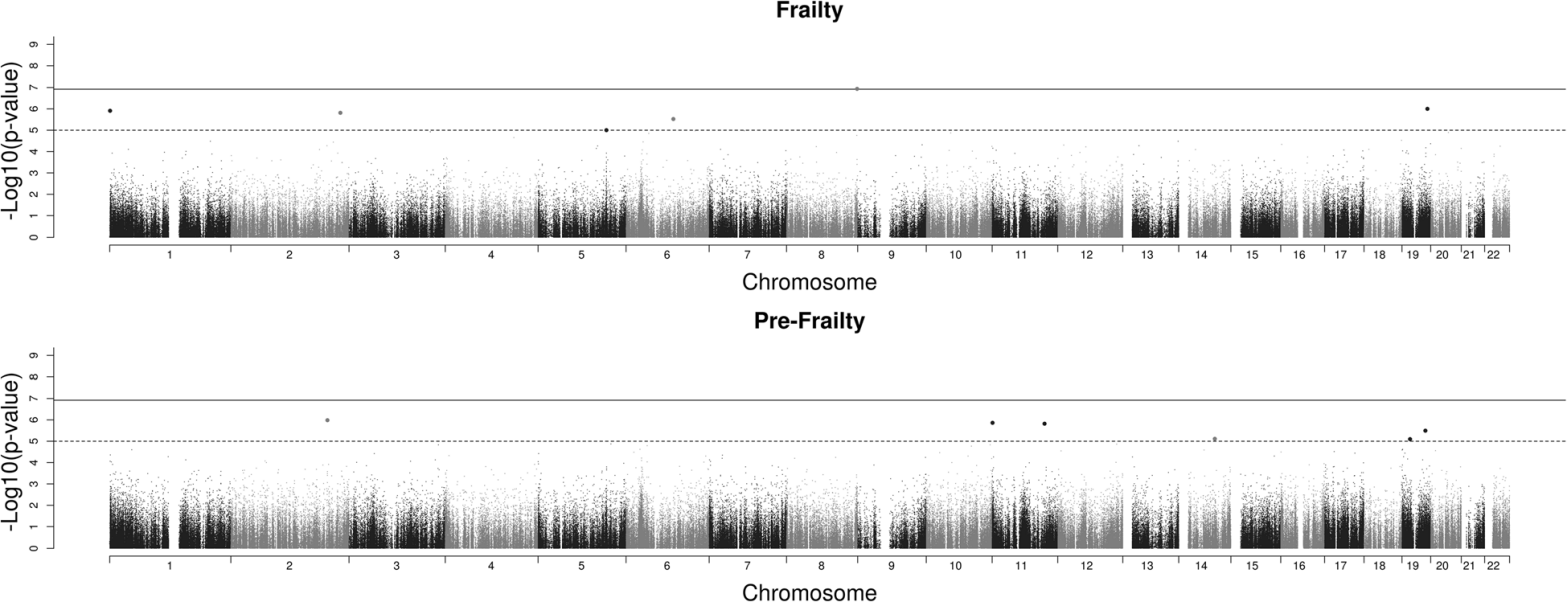
Manhattan plots for frailty versus no frailty and pre-frailty versus no frailty. The solid line represents a Bonferroni significance threshold

**Fig. 2:**
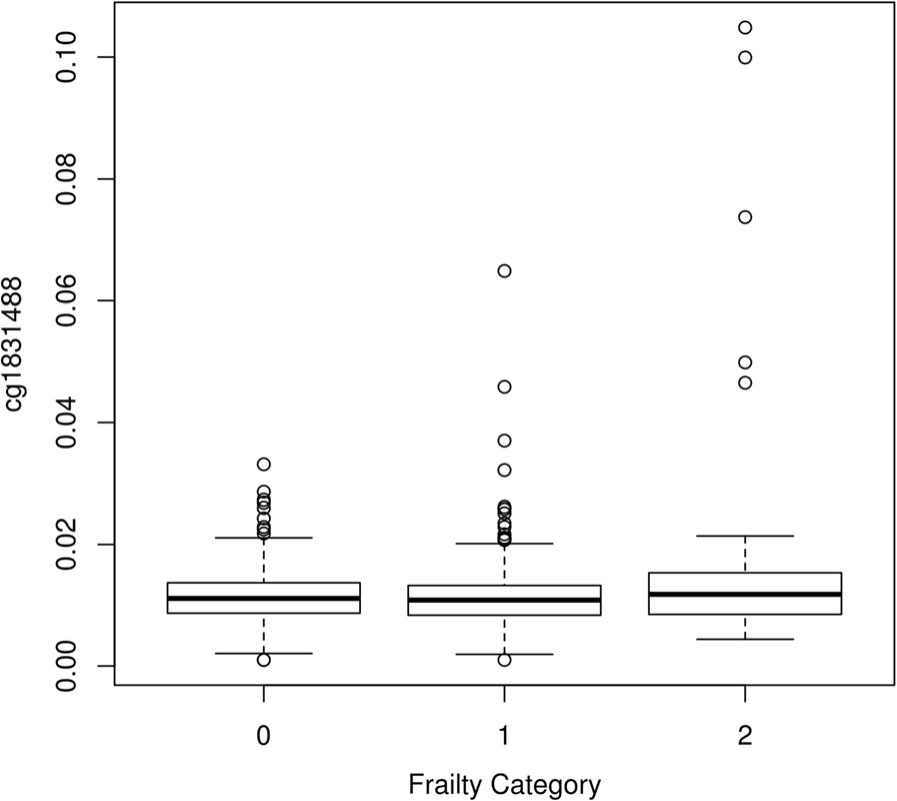
Boxplot of cg18314882 on chromosome 8 in the MAF1 gene according to frailty status (0 = not frail, 1 = pre-frail, 2 = frail)

Manhattan plots showing the *p* values for the CpGs for physical frailty status (frail versus not frail, and pre-frail versus not frail) are presented in Fig. 1. There was a single significant association in the EWAS comparing frail versus not frail: cg18314882 on chromosome 8 in the *MAF1* gene (*p* = 1.16 × 10–7): beta 0.0054 (SE 0.0010), indicating hypermethylation. Table 2 reports the local associations for CpGs within a 1601 base pair region (the rest of the CpG island). The significant site is an isolated result. Summary statistics for this CpG were mean 0.0118 (SD 0.007), minimum 0.001, maximum 0.105. Figure 2 shows a boxplot of this CpG by frailty status. A QQ plot of the *p* values for frail vs not frail is shown in the Additional file 1: Figure S1. We report the top 20 CpGs in the Additional file 1: Table S1. None of them are significant—the smallest *p* value is 0.012. The CpG that was significant in the EWAS comparing frail versus not frail was not significant in the EWAS comparing pre-frail versus not frail (*p* = 0.67).

**Table 2:** EWAS output for CpG sites in the same CpG island (chr8:145158467–145160068:Island) as the top signal (cg18314882)

Probe	Beta	SE	*T*	*p*
cg02621020	6.15E-04	0.001783	0.345243	7.30E-01
cg08825571	− 1.39E-03	0.000553	− 2.51254	1.22E-02
cg11538573	− 4.82E-04	0.000739	− 0.65216	5.15E-01
cg11883258	3.92E-03	0.004616	0.849065	3.96E-01
cg17170088	6.77E-04	0.000842	0.803668	4.22E-01
cg17176228	2.90E-04	0.000501	0.579033	5.63E-01
cg18314882	5.38E-03	0.001004	5.359905	1.16E-07
cg19517467	− 2.66E-04	0.0016	− 0.16609	8.68E-01
cg20573110	− 7.74E-06	0.001677	− 0.00461	9.96E-01
cg22260950	− 2.28E-04	0.001066	− 0.21425	8.30E-01
cg22861185	9.00E-04	0.000855	1.053655	2.92E-01
cg23119631	− 8.33E-04	0.001081	− 0.77111	4.41E-01

Table 3 shows relative risk ratios for being physically frail or pre-frail versus not frail according to the two measures of epigenetic age acceleration. Greater extrinsic age acceleration was associated with a slightly increased risk of being physically frail, but not of being pre-frail, in a model adjusted for age and sex: for a year increase in EEAA, the relative risk ratio (RRR) (95% CI) of being physically frail compared to being not frail was 1.06 (1.02, 1.10). This association was slightly attenuated after further adjustment for smoking status, alcohol intake, and number of chronic physical diseases, but remained significant: RRR (95% CI) was 1.05 (1.01, 1.10). When we adjusted for presence of cancer, high blood pressure, cardiovascular disease, stroke, diabetes, or arthritis as individual disorders, the association was very similar: RRR (95% CI) was 1.06 (1.01, 1.11). Greater intrinsic age acceleration was not significantly associated with risk of being physically frail or pre-frail. Relative risk ratios for the abovementioned analyses were unchanged in sensitivity analyses in which we additionally adjusted for white cell count and for technical variables related to the DNA methylation typing.

**Table 3:** Relative risk ratios (95% confidence intervals) for being physically frail or pre-frail according to epigenetic age acceleration at age 70

Epigenetic age acceleration measures, per year increase	Relative risk ratios (95% CI)		
Extrinsic epigenetic age acceleration	Not frail	Pre-frail	Frail
Adjusted for age and sex	Reference	1.02 (1.00, 1.04), *p* = 0.083	1.06 (1.02, 1.10), *p* = 0.006
Multivariable-adjusted^1^	Reference	1.02 (0.99, 1.04), *p* = 0.123	1.05 (1.01, 1.09), *p* = 0.023
Intrinsic epigenetic age acceleration			
Adjusted for age and sex	Reference	1.01 (0.99, 1.04), *p* = 0.248	1.03 (0.98, 1.08), *p* = 0.225
Multivariable-adjusted^1^	Reference	1.01 (0.99, 1.04), *p* = 0.316	1.02 (0.97, 1.06), *p* = 0.523

^1^Adjusted for age, sex, smoking status, alcohol intake, and number of chronic physical illnesses

## Discussion

In this cross-sectional survey of 791 men and women aged 70 years, epigenome-wide association study analyses found no widespread differences in methylation patterns between those who were physically frail and those who were not frail, as defined by the Fried phenotype. The proportion of methylation at a single CpG site (cg18314882 on chromosome 8 in the *MAF1* gene) was significantly different between those two groups. No such difference at any CpG site was found between those who were pre-frail and those who were not frail. These results suggest that blood DNA methylation is not a good biomarker for physical frailty. Older biological age as measured by extrinsic epigenetic age acceleration was associated with an increased risk of being physically frail, independent of potential confounding factors. Intrinsic epigenetic age acceleration was not associated with increased risk.

To our knowledge, there have been no previous EWAS of frailty status in later life. Here, we found that the proportion of methylation at one CpG methylation site (cg18314882 on chromosome 8 in the *MAF1* gene) differed between people who were physically frail and those who were not frail. There were no similar effect sizes for other CpGs within in the same region which would have increased the validity of our finding. Previous evidence in online databases suggests that this locus is unmethylated in every tissue, such that a beta of 0.02 was found in the blood, brain, omentum; see for example, Slieker et al. 2013 where these data are first described [30]. This limits the implication of this locus given that we found little indication that methylation was changed in relation to frailty. *MAF1* is a transcriptional repressor. Recent research has shown that it represses the expression of both pol III-dependent genes and certain RNA pol II-dependent genes that play a crucial part in oncogenesis, [31] and is important for the regulation of intracellular lipids [31, 32]. It is likely that *MAF1* has a diversity of physiological functions, but knowledge of its roles is still limited [33]. It is possible that it could influence risk of physical frailty via its role in lipid regulation and hence obesity. Obesity is an established risk factor for physical frailty [34, 35].

Sarcopenia, an age-related syndrome characterized by loss of skeletal muscle mass and strength, is a major contributor to physical frailty [36]. There is evidence from studies that measured methylation either in muscle tissue or in whole blood that methylation levels at some loci may help to explain variations in aging skeletal muscle mass [37, 38]. In a study comparing DNA methylation dynamics in skeletal muscle tissue from 24 young male adults and 24 older male adults, 5963 CpG sites were reported to be differentially methylated between the two groups; there was predominantly hypermethylation throughout the genome in the older group compared to the young group [37]. In a study of 1550 female twins that set out to identify genomic regions that were associated with skeletal muscle mass using methylation levels measured in whole blood, seven associations between methylation at CpG loci and skeletal muscle mass were discovered and replicated with a false discovery rate of less than 0.1 [38]. No association between individual CpG methylation sites and grip strength was found in a EWAS on the 27 k methylation array based on a sample of 172 female twins [39].

Our finding that greater extrinsic epigenetic age acceleration was associated with an increased risk of being physically frail is consistent with findings from a cross-sectional study of 1820 men and women aged 50–75 years [19]. In that study, greater epigenetic age acceleration (defined as the difference between predicted methylation age and chronological age) was associated with higher scores on a frailty index made up of 34 potential ‘deficits,’ such that that the frailty index increased by about 0.25% points per year of epigenetic age acceleration. There tends to be a moderate correlation between scores on a frailty index and physical frailty status as defined by the Fried phenotype [40], but they differ in that while the latter describes a specific clinical syndrome [41], the cumulative deficit model describes the general state or condition of an individual. The fact that greater epigenetic age acceleration has been shown to be associated with greater risk of being physically frail and of scoring higher on a more broadly defined frailty index [19] adds to the evidence that people who are frail are likely to be biologically older.

Intrinsic epigenetic age acceleration was designed to estimate “pure” epigenetic aging effects that are not influenced by differences in blood cell counts [15]. In the current study, it was moderately correlated with extrinsic epigenetic age acceleration (*r* = 0.38), and in contrast to the latter, it was not significantly associated with risk of being physically frail. Although both these epigenetic age acceleration measures are predictive of mortality [15], findings that intrinsic epigenetic age acceleration but not extrinsic epigenetic age acceleration predicts lung cancer [42] and is associated with being a centenarian [43] have led to the suggestion that it may capture a cell-type independent component of the aging process [15].

One potential limitation of our study is that of the 1091 individuals who took part in the survey, 791 (73%) could be included in the current study. Some individuals were missing data on the physical activity component of the Fried phenotype of frailty and some had missing methylation data. Another limitation is that we were only able to look at methylation markers in blood rather than in any other tissues in relation to physical frailty. Our EWAS findings may be due to either a genuine null association between blood-based methylation markers and physical frailty or a lack of statistical power in our current analyses.

In this cross-sectional survey of 70-year-old men and women, we found evidence that those who were biologically older, as indexed by greater extrinsic epigenetic age acceleration, were more likely to be physically frail. Future research will need to investigate whether epigenetic age acceleration plays a causal role in the onset of physical frailty.

## Additional file

Additional file 1: **Table S1.** EWAS output for the top 20 CpG sites from the analysis of frailty vs no frailty. **Figure S1.** EWAS-QQ plot of the *p* values for frail vs not frail. (DOCX 692 kb)

## Abbreviations

BMI: Body mass index
CpG: Cytosine-phosphate-guanine
DNA: Deoxyribonucleic acid
EEAA: Extrinsic epigenetic age acceleration
EWAS: Epigenome-wide association study
HADS-D: Hospital Depression and Anxiety Scale Depression subscale
IEAA: Intrinsic epigenetic age acceleration
IQR: Interquartile range
LBC1936: Lothian Birth Cohort 1936
SD: Standard deviation
