# Supplementary material for: DNA methylation and the epigenetic clock in relation to physical frailty in older people: the Lothian Birth Cohort 1936
Source: Clin Epigenetics. 2018 Aug 3;10:101. doi: 10.1186/s13148-018-0538-4 (PMC6091041; doi:10.1186/s13148-018-0538-4)

**Supplementary table: EWAS output for the top 20 CpG sites from the analysis of frailty vs no frailty**

| **Probe** | **Chromosome** | **Position (GRCh37/hg19)** | **Beta** | **SE** | **P** |
| --- | --- | --- | --- | --- | --- |
| cg18314882 | 8 | 145159734 | 0.005 | 0.001 | 1.16E-07 |
| cg21910821 | 19 | 52407881 | 0.014 | 0.003 | 9.98E-07 |
| cg08167039 | 1 | 762232 | 0.009 | 0.002 | 1.23E-06 |
| cg00698413 | 2 | 225266656 | 0.025 | 0.005 | 1.53E-06 |
| cg27299725 | 6 | 97345805 | 0.005 | 0.001 | 2.99E-06 |
| cg03335262 | 5 | 140536920 | 0.045 | 0.010 | 9.95E-06 |
| cg18782991 | 5 | 140479979 | 0.033 | 0.007 | 1.00E-05 |
| cg10928348 | 7 | 71877113 | -0.018 | 0.004 | 1.06E-05 |
| cg24377437 | 6 | 142047924 | -0.026 | 0.006 | 1.09E-05 |
| cg24527262 | 3 | 167098170 | 0.010 | 0.002 | 1.18E-05 |
| cg14160807 | 20 | 36156231 | 0.003 | 0.001 | 1.26E-05 |
| cg25805482 | 10 | 50397618 | -0.016 | 0.004 | 1.35E-05 |
| cg27612364 | 6 | 46620935 | 0.011 | 0.002 | 1.40E-05 |
| cg21925025 | 8 | 144508684 | 0.039 | 0.009 | 1.73E-05 |
| cg27386292 | 4 | 141295273 | 0.008 | 0.002 | 2.20E-05 |
| cg00461905 | 18 | 77315962 | -0.004 | 0.001 | 3.01E-05 |
| cg16061436 | 1 | 206910867 | -0.075 | 0.018 | 3.28E-05 |
| cg17835016 | 13 | 113784082 | 0.045 | 0.011 | 3.32E-05 |
| cg03772072 | 6 | 35392381 | -0.014 | 0.003 | 3.49E-05 |
| cg12903638 | 2 | 210636350 | 0.020 | 0.005 | 3.58E-05 |

Full summary statistics are available at the following link http://www.ccace.ed.ac.uk/node/335

**Supplementary figure: EWAS - QQ plot of the p-values for frail vs not frail**


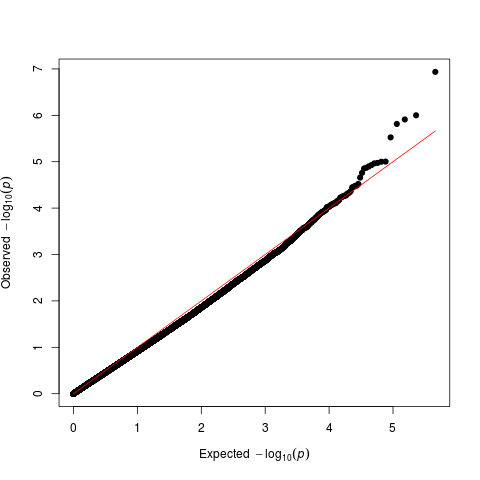

Supplement: Supplementary file 1 — Table S1. EWAS output for the top 20 CpG sites from the analysis of frailty vs no frailty. Figure S1. EWAS-QQ plot of the p values for frail vs not frail. (DOCX 692 kb) [file 13148_2018_538_MOESM1_ESM.docx]
